# Senses in New-Born Infants

**Published:** 1883-07

**Authors:** 


### SENSES IN NEW-BORN INFANTS.

In a recent inaugural dissertation Dr. Genzmer discusses the activity of the senses in new-born infants. *Inter alia,* he says the sense of touch is developed from the earliest period, and reflex actions are readily excited by the slightest stimulation of the nerves of touch, especially that of the face, then of the hands and soles of the feet. The feeling of pain is but slowly developed, and is only clearly shown after four or five weeks, before which time infants do not shed tears. Smell and taste are not distinguishable in infants. Hearing is perceptible in the first, or at most, the second day of life. New-born infants are so sensitive to light that they will turn the head to follow a mild light, while if a strong glare be suddenly thrown on the eye squinting is induced, and even convulsive closure of the lids. After a few days the child will follow the motion of various objects by movements of its head. Between the fourth and fifth week the convergence of the pupils and co-ordination in vision are perceptible. A distinct perception of color does not exist under four or five months; before then it is quantity rather than quality of light that is recognized.—*English Mechanic.*

